# Pneumothorax with Effusion, Post Mortem, &c.

**Published:** 1853-07

**Authors:** Ira E. Oatman

**Affiliations:** Sacramento City, California


ART. III.—*Pneumothorax with Effusion: Post mortem, etc.* By IRA E. OATMAN, M. D., of Sacramento City, California.

MR. A., æt. about 33, sanguine—nervous temperament, of replete habit and a free liver, had been afflicted with some disease of the chest since the great conflagration of this city, in November last—having been subject to a chronic cough for many years. He spent the winter in San Francisco on account of the fire and flood in this city. On his return, he was suddenly attacked with dyspnœa; which nearly proved fatal in a few hours. He was treated by another physician for Hydrops Pericardii, with some improvement for a month. A stupid quack being allowed to prescribe, the attending physician withdrew from the case; and I was called on the 6th of March last. I found the patient sallow and emaciated, witn œdema of the extremities.

He had colliquative diarrhœa, hectic fever and night sweat, the pulse 90 to 100 per minute. The epigastric and hypochondrial regions much distented, elastic, tender and dull on percussion. The left side of the chest enlarged, immovable, the intercostal spaces protruding and extremely resonant on percussion down to the 6th rib, in the erect position. Slight dullness in the superior posterior region. Auscultation revealed no respiratory sound of that side; but the cavernous sound of the voice. Succussion produced the splashing sound, followed sometimes by the metallic tinkling. The respiration of the *right* side hurried and puerile. The right mammary region dull on percussion. The impulse and sound of the heart beneath the right nipple. Mucous rale in the right subclavicular region—the expectoration being muco-purulent. The treatmeat was tonic and astringent, with an anodyne at bed-time and the Pot. Iodide. Later in the disease, he took the Cod Liver Oil.

The Diagnosis was Pneumothorax with effusion and hypertrophy, or abscess of the liver. He died comatose, on the 22d of the same month. *Post mortem*, 30 hours after death, in presence of six other physicians. A small puncture being made into the left side of the chest, a great quantity of (apparently) sulph. hydrogen gas escaped with a hissing sound.

The left pleural sac contained about a gallon of purulent fluid about the consistence of milk. The costal pleura ulcerated and gone,—leaving some of the ribs bare. The left lung compressed to less than one fourth its natural size, was hepatized, tuberculous and impervious to air. The right lung had tubercles in the superior portion. The heart and pericardium were natural but with the mediastinum, were pressed into the right side,—the heart being beneath the right nipple.

The liver was pale and hypertrophied to twice its natural size. The other viscera not examined.

I proposed to practice paracentesis thoracis, but, on being told he could not recover, his friends declined having it done.

This had undoubtedly been a case of neglected, or maltreated, pleuropneumonia, with tuberculosis of the left lung. That the gas, or air with which the cavity was filled, was not from the decomposition of the effused fluid, but that it passed through the lung, I was fully convinced by the manner of its appearance.

Sacramento, May 10, 1853.

